# 3D Inkjet Printing of Complex, Cell-Laden Hydrogel Structures

**DOI:** 10.1038/s41598-018-35504-2

**Published:** 2018-11-20

**Authors:** Andrea Negro, Thibaud Cherbuin, Matthias P. Lutolf

**Affiliations:** 1 Laboratory of Stem Cell Bioengineering, Institute of Bioengineering, School of Life Sciences and School of Engineering, École Polytechnique Fédérale de Lausanne (EPFL), Lausanne, CH-1015 Switzerland; 2 Institute of Chemical Sciences and Engineering and School of Basic Science, EPFL, Lausanne, CH-1015 Switzerland

## Abstract

Inkjet printing is widely considered a promising strategy to pattern hydrogels and living cells into three-dimensional (3D) constructs that structurally resemble tissues in our body. However, this approach is currently constrained by the limited control over multi-component deposition: the variable droplet ejection characteristics of different bioinks and dispensing units make synchronized printing very challenging. This invariably results in artificial tissues that lack the complexity and function of their native counterparts. By careful optimization of the printing parameters for two different bioink formulations, here we report the inkjet-based 3D-patterning of hydrogels according to relatively complex blueprints. 3D printing of bioinks containing living cells resulted in high-resolution, multi-component living constructs. Finally, we describe a sacrificial material approach to inkjet print perfuseable channels for improved long-term cultures of larger samples. We believe that this work provides a foundation for the generation of more complex 3D tissue models by inkjet printing.

## Introduction

Tissues are multi-component entities harboring various cell types, embedded in cell- and tissue-specific extracellular matrix (ECM) hydrogels, with a characteristic 3D architecture that is crucial for tissue function^1^. Furthermore, tissues are dynamic entities in which cells self-organize by migrating, proliferating, specializing and actively remodeling their ECM^2^. Recapitulating and inducing this spatial and temporal tissue complexity outside of the body to an extent that gives rise to some functionality of a tissue represents one of the most exciting challenges of modern tissue engineering^3,4^.

Recapitulating spatiotemporal tissue complexity *in vitro* entails two key challenges. The first is to formulate biomaterials that can mimic the physiological extracellular milieu to some extent and, as such, promote physiological cell behaviors and ultimately cellular self-organization into tissues^2,5,6^. Second, if self-organization and morphogenesis is limited, as is the case for most cell types and *in vitro* culture systems, it may be necessary to arrange tissue building blocks in 3D in an *in vivo*-like manner^7–9^. Inkjet printing platforms are potentially highly efficient tools to recreate the 3D architecture of a tissue using computer-aided deposition^7,10,11^ Drop-on-demand (DOD) systems, for example, grant the possibility of contactless depositing a wide range of biomaterials in successive layers to generate 3D structures^12,13^. Indeed, DOD systems offer the possibility of multi-component dispensing^14^, and previous studies have shown the use of multiple nozzles, typically for printing ink precursor and subsequent rapid deposition of a cross-linker^15,16^. However, even though multi-component ink printing has already been used with other techniques such as extrusion^17^, and low-resolution cell-containing printouts have been reported^18^, the use of multiple inks, and thus the possibility to print more realistic tissue-like constructs, has remained elusive^11^. Moreover, compared to other approaches, DOD techniques may offer advantages in terms of the number of printable materials, control over the volumes released, and the spatial resolution^19^. However, despite exciting pioneering work on biomaterials and cell printing using DOD^20–23^, we believe that the potential of this technique for tissue engineering has not been fully exploited.

We had previously identified two critical conditions for inkjet printing of high-resolution 3D hydrogel structures, namely *(i)* dispensed droplets must retain a 3D structure and *(ii)* gelation must be very rapid^10,24^. To fulfill these requirements, we had used alginate, an anionic polysaccharide that cross-links in milliseconds via calcium complexation^24,25^. To 3D-print alginate structures, we had induced cross-linking of printed droplets by delivering calcium ions from a hydrogel substrate that stored large amounts of calcium. Although this work represented an important step forward towards DOD-based bioprinting, the controlled high-resolution 3D-printing of different bioinks has not yet been reliably achieved, because bioinks differ in terms of ejection characteristics such as viscosity and surface tension. Here, we optimized printing parameters for different cell-containing bioink formulations, resulting in high-resolution multi-component living constructs. This approach should be useful for the generation of 3D tissue models for basic biological studies and disease modeling in drug discovery and diagnostics.

## Results and Discussion

### Multiple Inkjet Nozzles Alignment and Calibration

**Figure 1:**
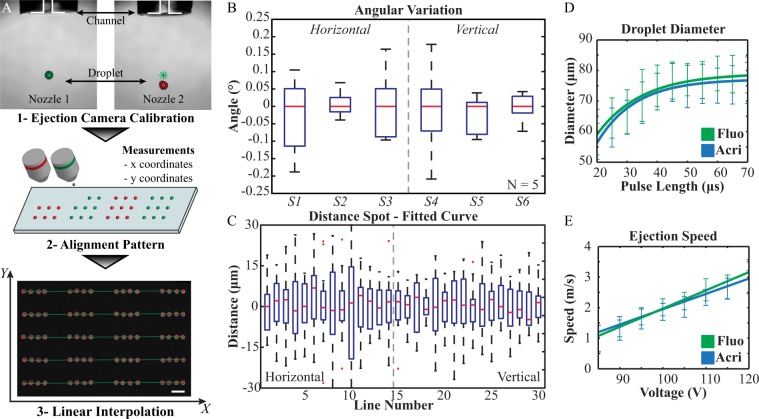
Evaluation of nozzle alignment and synchronization of ejection performance. (**A**) Step 1. Ejection adjustment to obtain homogeneous ejection states (channels diameter 70 μm). Step 2. Dispensing of linear double color arrays. Step 3. Imaging and interpolation. Red asterisks represent spot positions. Green lines are the fitting curves (scale bar 500 μm). (**B**) Angular coefficients, representation of parallelism between the printed lines. (**C**) Distribution of distances between detected spots and interpolation curves (red asterisk and green lines in A), line by line across all samples. (**D**) Diameter variation under pulse-length influence: Acri-ink (blue), Fluo-ink (green). (**E**) Speed versus voltage function is reported for both systems: Acri-ink (blue), Fluo-ink (green).

To create a complex 3D pattern composed of different cells and biomaterials, it is crucial that multiple nozzles to dispense inks are well aligned. We used a simplified model system composed of alternating spots deposited by two independent dispensing units of a commercial inkjet printing system (Fig. 1). The accuracy in the spatial alignment was assessed by microscopy and image analysis and characterized by measuring the correlation between printouts and blueprints in a simple pattern composed of linearly arranged spots deposited by each dispensing unit in an alternating fashion (Fig. 1A). To avoid variability introduced by ink differences, we loaded both nozzles with the same ink. Moreover, the dispensing parameters (voltage and pulse-length) were set to the same values for both dispensing units, producing steady ‘ejection states’ at both dispensers. Our evaluation was based on three indexes that would indicate the quality in the spatial alignment of two inkjet-printed drop populations. *(i)* The R^2^ coefficients from interpolation results showed very high correlation between blueprint and printouts (R^2^ = 0.997 ± 0.002). *(ii)* The angular deviation (*i.e*. the angle between the droplet trajectory and the axis of the nozzle) of different samples (Fig. 1B) was affected by minimal deviation within the different line populations (±0.2°). *(iii)* The distances between the printed spots and the trend line (Fig. 1C) was characterized by an average value equal to 0 and a standard deviation equal to 10 μm (corresponding to less than 10% of spot size). These results led us to conclude that the geometric alignment of the dispensing units was appropriate for multi-component patterning.

In addition to highly precise spatial alignment of printed droplets, the homogeneity of droplet ejection among nozzles is equally important for high-quality 3D bioprinting. To this end, we first evaluated the ‘printability’ of two fluorescently labeled (aminoacridone and aminofluorescein) alginate-based model bioinks (termed ‘Acri-ink’ and ‘Fluo-ink’, respectively) that were formulated based on work by Braschler^26^ (Supplementary Information Figure S1). The main component of both inks is 0.8% w/v alginate (molecular weight circa 100 kDa) supplemented by 0.05% w/v Pluronic PE6800 to reduce surface tension. The printability of these two bioinks was evaluated by calculating the Z number defined as follows^27^:

1 $$Z=\frac{\sqrt{\gamma \cdot \rho \cdot a}}{\eta }$$

where γ, ρ, η and *a* refer to fluid surface tension, density, viscosity and characteristic length (nozzle diameter, 70 μm), respectively. The optimal printable range corresponds to Z between 1 and 10. Outside of this range printing could be affected by satellite droplet formation (Z > 10) or by a viscous dissipation preventing droplet ejection (Z < 1)^27^.

The characteristics of the bioinks are reported in Table 1. Accordingly, the Acri-ink was found to be characterized by a Z (11.66 ± 0.41) slightly outside of the optimal range due to differences (p < 0.001) in both viscosity and surface tension when compared to Fluo-ink. Indeed, during the empirical printability evaluation, Acri-ink showed a tendency to form satellite drops when high-energy states were used to drive the ejection. However, it was nevertheless possible to identify stable ejection states within the investigated parameter range for Acri-ink.

**Table 1:** Measurements of Acri-ink and Fluo-ink features to evaluate their theoretical printability according to the Z calculation. All parameters are statistically independent when comparing the two ink formulations.

	Density [kg/m^3^]	Surface tension [N/m]	Viscosity [kg/ms]	Z
Acri-ink	979.1 ± 59.6	0.0537 ± 0.0005	0.005 ± 0	11.66 ± 0.41
Fluo-ink	1003.4 ± 4.4	0.0546 ± 0.0008	0.009 ± 0	6.99 ± 0.06

Next, we assessed the empiric printability of Acri-ink and Fluo-ink using a Microdrop Autodrop inkjet printer. We specifically investigated two indexes related to the droplet ejection characteristics, namely the droplet size and ejection speed (Supplementary Information Figure S2). Droplet size directly correlates with the dimension of the printed spot on a substrate. When multiple inks are dispensed, the size of their spot has to be uniform; therefore, droplet size must be independent of the ink composition. The droplet dimensions are crucial to the print and have to be taken into consideration when designing blueprints for desired 3D constructs. Additionally, the ejection speed is crucial for setting up reliable multi-component dispensing; the speed of dispensing units must be homogeneous to guarantee proper patterning.

Droplet diameter and ejection speed were modulated by adjusting the voltage and pulse-length on each dispensing unit. Using image analysis, we assessed 88 combinations of voltage (85–120 V, 5 V steps) and pulse-length (20–70 μs, 5 μs increments) for each bioink. We found an exponential relationship between pulse-length and droplet diameter for both bioinks (Acri-ink: R^2^ = 0.962, Fluo-ink: R^2^ = 0.969) (Fig. 1D, Supplementary Information Figures S3 and S4). Interestingly, an asymptotic value in droplet diameter was obtained for both inks (77 μm and 79 μm for Acri-ink and Fluo-ink, respectively), representing the limit in droplet size that can be generated with a given nozzle (here, 70 μm). Furthermore, for both bioinks we found a linear relationship between the voltage and ejection speed (Acri-ink: R^2^ = 0.996, Fluo-ink: R^2^ = 0.989) (Fig. 1E, Supplementary Information Figs S3,4). This process was later used to synchronize alternative formulation of the inks.

### Inkjet Printing of Complex Cell-laden Structures

**Figure 2:**
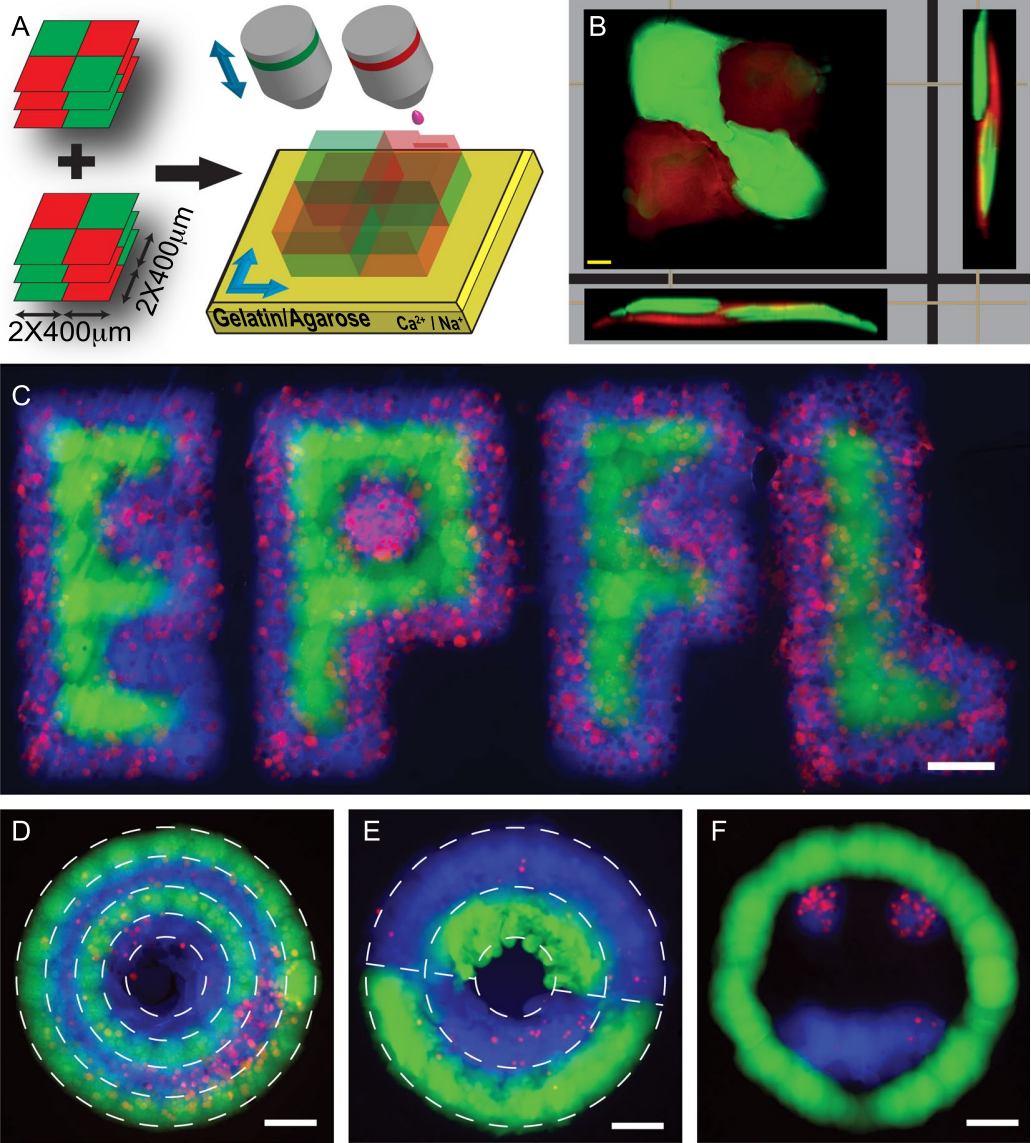
Multicomponent patterning of cell-containing hydrogels. (**A**) Schematic representation of 3D checkerboard composed of two patterns (overprinted 3 times each): Fluo/Acri (top) and Acri/fluo (bottom). (**B**) Bioprinted 3D checkerboard. The top view represents the single layer fashion, while side and front views show the vertical alternation of colors (Scale bar: 100 μm). Acri-ink was rendered red to increase the contrast. (**C**–**F**) Patterns obtained by printing Fluo-ink (green) and Acri-ink (blue) containing Tomato NIH 3T3 fibroblasts (red). (**C**) University logo, inner section: double line letters; outer stroke: single line. (**D**) Concentric circles were printed by alternating Fluo-ink and modified Acri-ink twice. (**E**) In partial circles pattern, two-lined circles were obtained alternating the two inks. White dashed lines represent the bluprints (in **D**,**E**). (**F**) ‘Smiley face’ to demonstrate the flexibility of our approach (**C**–**F**: scale bar 200 μm).

Having optimized the printing parameters for the two bioink formulations, we next sought to inkjet-pattern these two bioinks into 3D objects. To assess the quality of 3D patterning by confocal microscopy, we first chose the conceptually simple pattern of a 3D checkerboard (Fig. 2A,B). A comparison between the blueprint and the patterned two-component hydrogel structures revealed that the latter were approximately 5% smaller in length (Supporting Figure S5) and approximately 10% higher than the prediction (119.2 ± 4.7 μm vs. 108 μm). No noticeable difference between the measurements taken along either horizontal or vertical direction was found, implying the absence of any sort of anisotropy. These results show that our optimized printing strategy facilitates high-resolution 3D hydrogel patterns that correctly reproduce the blueprints.

The potential of our 3D multicomponent hydrogel patterning approach was further tested by producing more complex single layer structures using two different inks and by introducing living mammalian cells (Tomato NIH 3T3 fibroblasts, 3E6 cells/mL) into one of the bioinks (Acri-ink). The ejection performances of the new ink were tested according to the above-described methodology showing no difference when compared with Acri-ink without cells. Figure 2C–F shows the printouts and relative blueprints (white dashed lines), revealing high precision in multi-component patterning, even in the presence of cells in Acri-ink (red dots). Notably, the flexibility of our printing strategy allowed us, in later iterations, to design and pattern a wide variety of 3D shapes of several centimeters long and millimeter high with a precision of approximately 100 μm. This reliability was maintained even when cells were present in one of the bioinks. Importantly, a live/dead assay 4 h after bioprinting revealed that on average 92% (±5%) of bioprinted cells (NIH 3T3 fibroblasts added to the alginate-based inks at a density of 3E6cells/mL) remained alive.

### Inkjet-based Fabrication of Microfluidic Networks

**Figure 3:**
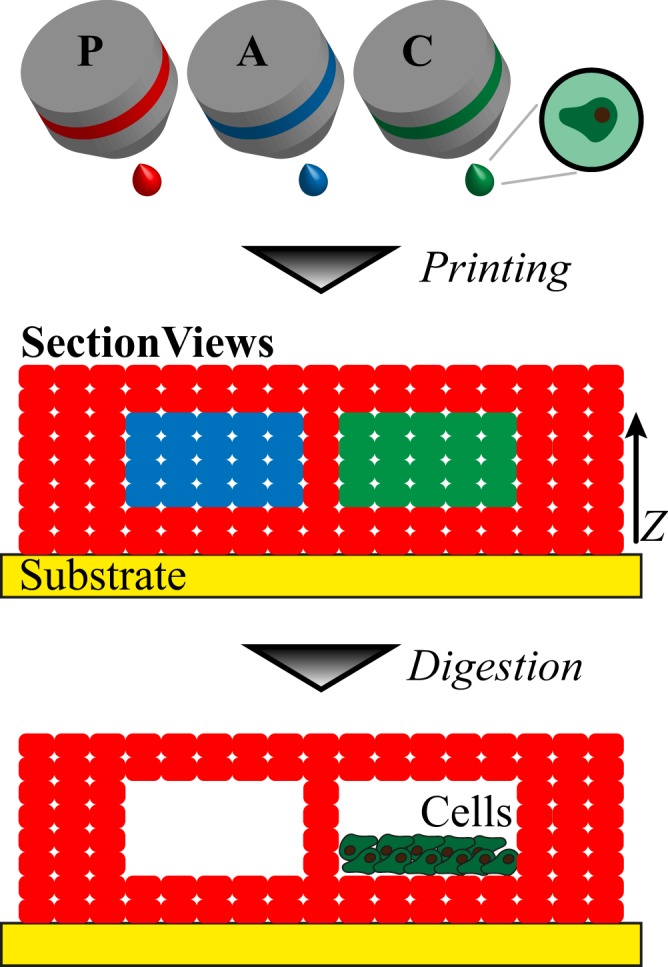
Sacrificial layer approach to form hollow cell-containing channels inside 3D-bioprinted constructs. Side section representation of permanent ink (P, red), sacrificial Acri-ink (A, blue). The sacrificial ink can be modified to host mammalian cells (C, green). Step 1: drop-by-drop printing of the multiple components; step 2: alginate digestion triggering cell sedimentation at the bottom of the channel.

**Figure 4:**
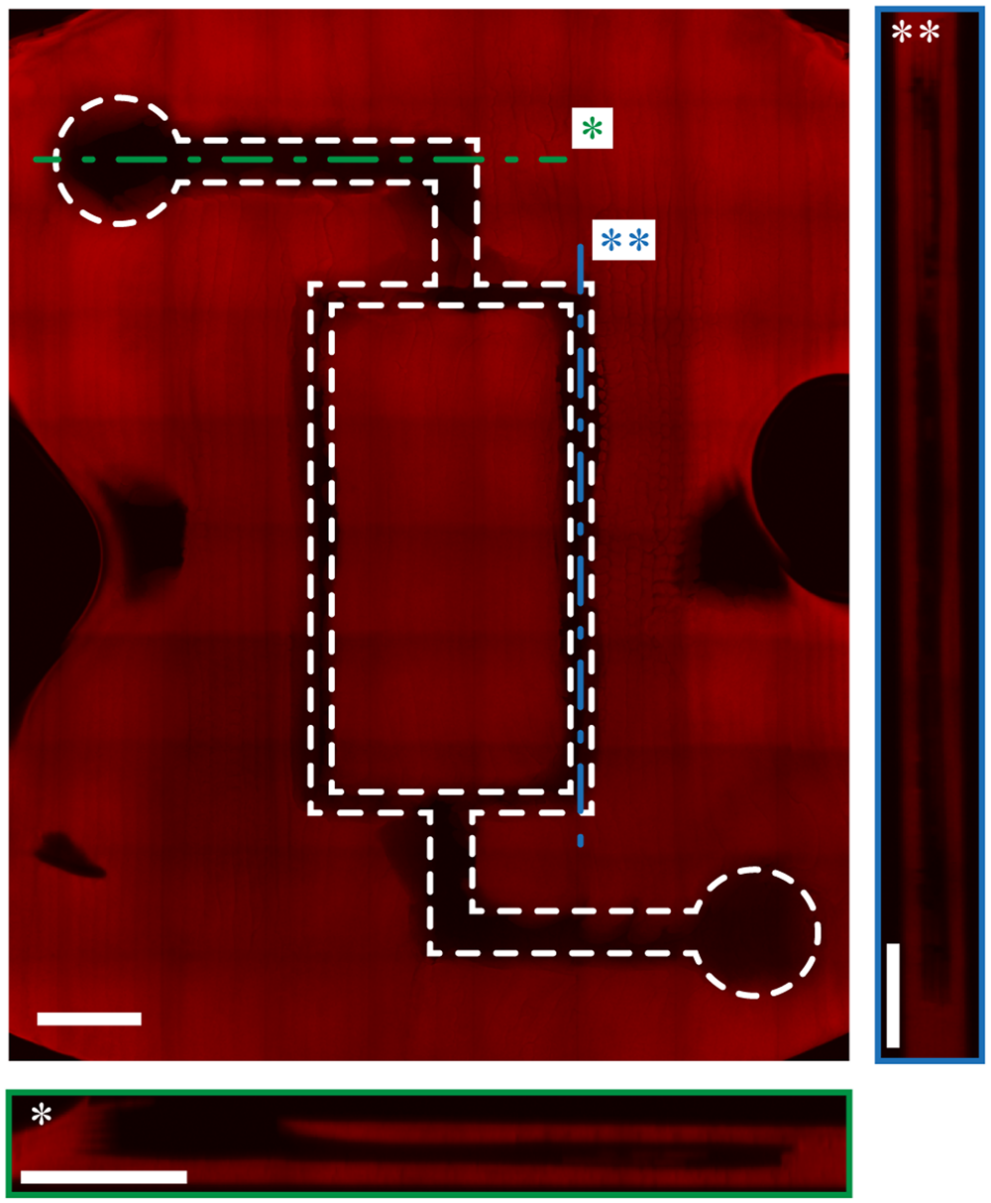
A bioprinting hydrogel-based microfluidic chip. The sacrificial material approach shown in Fig. 3 was used to pattern microfluidic networks. Longitudinal confocal section imaging of microfluidic network produced by the sacrificial layer technique. Side section of the main channel (green*). Side section of one branch (blue**). Scale bar: 1 mm.

In order to solve the diffusion limitation within larger tissue constructs, we designed and tested a sacrificial material bioprinting approach. Our methodology is based on different alginate-based inks and their selective digestion by alginate lyase enzyme^28^ to generate perfusion-ready networks within the printouts (Fig. 3). We first printed several layers of permanent ink (corresponding to Fluo-ink), followed by a pattern of layered sacrificial ink (corresponding to Acri-ink) and permanent ink, and finally topped by several layers of permanent ink. Indeed, by alternating alginate lyase-digestible (Acri-ink) and non-digestible (Fluo-ink) bioinks in multi layers, it was possible to obtain complex 3D printouts in the form of microfluidic networks (Fig. 4). Subsequent exposure to alginate lyase removed the sacrificial material, resulting in hollow, connected fluidic networks as demonstrated by confocal microscopy. It should be noted that the size of the sacrificial pattern may be adapted by printing multiple droplets alongside or on the top of the other leading them to fuse. Moreover, by overlapping several layers of sacrificial ink containing cells and proceeding to their digestion, we were able to enhance their local density and obtain a higher cell mass through sedimentation. Therefore, we believe that this approach may also overcome the problem of limited cell density of common bioprinting approaches^11^.

### Bioprinting of 3D Cell-Laden Structures

**Figure 5:**
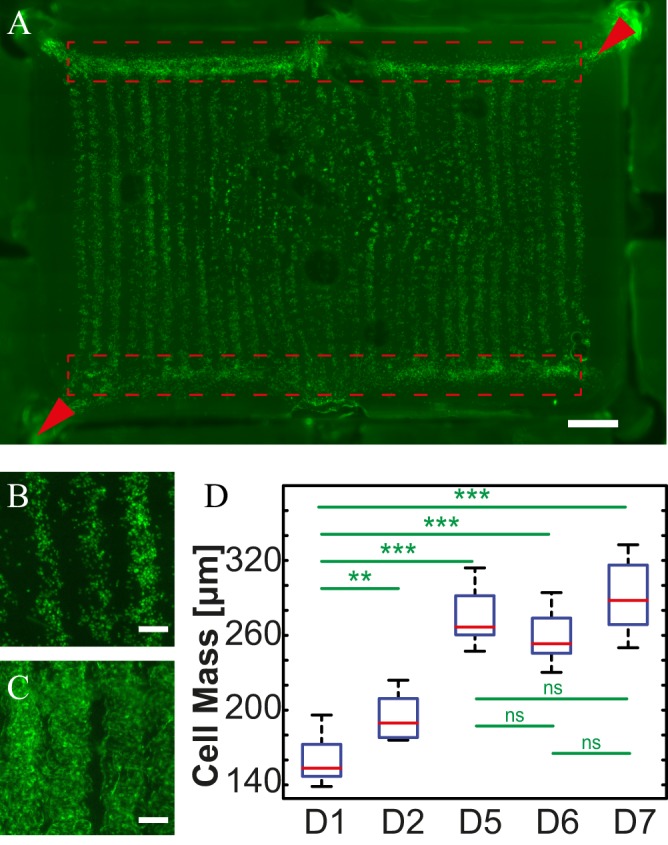
A bioprinted cell-laden network. (**A**) Printout after the digestion and 1 h of perfusion. Dashed-line rectangle outline the distribution channels, arrowheads indicate the perfusion direction. (**B**) On day 1 cells cluster at the bottom of the channels. (**C**) On day 7, cells grown under perfusion have fully populated the channel structure. (**D**) Quantification of changes in cell mass as a function of time. Scale bars: 1 mm (**A**), 200 μm (**B**,**C**).

We next tested whether living mammalian cells could be inkjet-printed into 3D constructs via our approach. To increase the biological activity of the alginate-based bioinks, we modified them with a biomimetic, poly(ethylene glycol) (PEG)-based hydrogel network. Specifically, we used a previously published^29^ matrix metalloprotease (MMP)-sensitive PEG gel (termed TG-PEG) that can be crosslinked via the active transglutaminase (TG) factor XIII and further contains an RGD (Arg-Gly-Asp) peptide ligand (50 μM) to promote cell adhesion through integrin binding. For multi-component cell printing, we used two bioinks: alginate supplemented with TG-PEG (termed ‘ECM-ink’) and alginate supplemented with GFP-expressing C2C12 muscle progenitor cells as our cell model (‘Cell-ink’). These two inks were 3D patterned into a ladder-like microfluidic network (Supplementary Information Figure S6) comprised of 4 layers of Cell-ink separated by 3 layers of ECM-ink. The microfluidic network was designed to feature inlet and outlet structures to allow subsequent perfusion upon digestion of the Cell-ink (Fig. 5A). In order to establish a perfusion flow within the 3D-printed microfluidic network, we utilized a support structure produced in PDMS^24^.

Cell behavior in bioprinted constructs was assessed by fluorescent microscopy over one week in culture, demonstrating a clear increase in cell mass over time (Fig. 5B–D). Importantly, the establishment of medium perfusion through the network was found to be essential for supporting cell proliferation and tissue growth.

Taken together, this work demonstrates the possibility of aligning and synchronizing multiple independent inkjet units to generate 3D multicomponent hydrogel structures. The good patterning resolution, together with the remarkable cell compatibility and the sacrificial layer technique, opens up exciting perspectives for tissue engineering.

## Methods

### Materials

All reagents were purchased from Sigma-Aldrich AG (Buchs, Switzerland). An Autodrop platform (Microdrop GmbH, Norderstedt, Germany) was used as the robotic dispenser. The printer was equipped with two MD-K-130 nozzles characterized by an outlet diameter of 70 μm. The fluorescent alginates were prepared as described^26^.

### Nozzle Alignment and Calibration

Robot calibration was based on relative nozzle position in front of the dedicated ejection camera. The validation ink was prepared by diluting glycerol to 15% w/v in water. Relative positions of nozzles were fixed upon ink loading. Once drop ejection was stabilized for both nozzles (same diameter and ejection speed), camera positions were refined and the software calculated the relative distance between the nozzles. The validation was performed by assessing the alignment of spots on a straight line printed by alternating the two dispensing units. The testing pattern consisted of ten lines (five horizontal and five vertical). Each line was divided into four segments, obtained by aligning four spots. The distance between spots was set at 300 μm, while both the line and segment separation were equal to 1 mm. The first dispensing unit was used to print each odd numbered segment, while the second was set to print the even numbered sections of each line. After dispensing, the printed samples were imaged on an Axio Observer inverted microscope (Zeiss, Thornwood, U.S.A.). Metamorph software (Molecular Devices, Sunnyvale, U.S.A.) was used to control the microscope and to reconstruct images. ImageJ (NIH, U.S.A.) and Matlab (MathWorks, Natick, U.S.A.) permitted image processing, data analysis and statistics.

### Alginate-Based Bioinks

Water-based ink compositions were obtained by mixing fluorescently modified alginate (stocks: 2% w/v aminoacricone-alginate or 2% w/v aminofluorescein-alginate) and pluronic PE6800 (stock: 2% w/v) obtaining bioinks containing 0.8% w/v alginate and 0.05% w/v surfactant. When cells were present (Cell-ink), we added them to achieve a final concentration of 3E6 cells/mL and raw-alginate was used when fluorescent imaging was involved.

### ECM-ink

A hybrid hydrogel system was used ‘ECM-ink’. This system is composed of two previously described gel networks, namely alginate and a synthetic PEG hydrogel that is crosslinked by the activated transglutaminase Factor XIII (FXIIIa)^29^. The hybrid gel network is composed of a blend of alginate (0.5% w/v) TG-PEG (3%w/v) with an enzyme concentration of 30 U/mL. To prevent crosslinking prior to 3D bioprinting, calcium was removed and EDTA 0.66 mM was added.

### Ejectability Potential

Ejectability was theoretically assed by the dimensionless index, the Z number, as reported by Derby^27^. *(i)* Surface tension was directly measured by the du Noüy ring method by a Sigma 703 tensiometer (KSV Nima/Biolin Scientific, Espoo, Finland). *(ii)* Density was estimated by comparing ink and water densities, weighing equal volumes of each liquid. *(iii)* Viscosity was estimated by a Micro-Ostwald viscometer (SCHOTT Instruments GmbH, Mainz, Germany). For empirical evaluation of droplet formation, a screen of over 88 droplet-generating parameter sets was performed. Voltage and pulse-length defined each dispensing state. The first was varied among the range 85–120 V with 5 V increments, while pulse-length was set in the range 20–70 μs (5 μs step). Frequency was set to 100 Hz during the entire test. The ejection camera acquired two images of each ejection state, characterized by different lag times compared to excitation pulses. Image analysis was performed in ImageJ, and we analyzed the data with Matlab, evaluating ejection speed and droplet diameter. A scheme of measurement is reported in Supplementary Information Figure S2.

### 3D Patterning of Multi-Hydrogel Constructs

A 3D checkerboard was implemented using Autodrop software. Small 2 × 2 checkerboards were designed with side lengths equal to 400 μm/cell. The first color motif (Fluo/Acri) was overprinted three times, and then the dual pattern (Acri/Fluo) was overprinted another three times. After printing, samples were immersed in water and stored at 4 °C. Confocal microscopy was used to investigate the constructs by “Z-stack mode,” and 3D stacks were acquired. We measured geometric features in Imaris (Bitplan, Zurich, Switzerland): cell side length, total and partial heights. Data analysis and statistics were performed using Matlab.

### Arbitrary Patterns

University logo, four concentric circles, double-color rings and smile (all circular designs were 1 mm external diameter) were programmed via Autodrop software. Printing was performed against a calcium containing hydrogel to promote alginate cross-link as previously reported^24^. The samples were imaged under an Axio Observer inverted fluorescent microscope. Image processing was then performed in ImageJ.

### Microfluidic Patterns

Both presented microfluidic pattern (with and without cells) were design to obtain a final 12 × 8 × 1 mm structure. The printing was performed against a calcium containing hydrogel substrate to promote the cross-linking reaction as previously reported^24^. Bifurcating channels (Fig. 4) consisted of circular element of 1 mm in diameter, both linked with 400 μm wide channel (L-shape), themselves connected by two 200 μm channels (central rectangular shape). Confocal microscopy was used to investigate the constructs by “Z-stack mode,” and 3D stacks were acquired. The cell-laden network (Fig. 5) consisted of two 400 μm distributions channel connected by thirty-two 100 μm secondary channels. Secondary channels were spaced by 300 μm bulk ECM-ink (Supplementary Information Figure S6). Perfusion was performed in a PDMS bioreactor chamber as described by Pataky *et al*.^24^. After lyase treatment, a peristaltic pump was connected to the chamber. A pulsatile flow of alternating 1 s flow at 5 μl/min and 9 s pause was performed during the entire culture phase. Cell growth across the printout was assessed by microscopy and image analysis over a period of 7 days. In both patterns, alginate lyase digestion was achieved by incubation for 2 h at 1 U/mL in cell media at 37 °C in standard cell incubator.

### Live/Dead Assay

Calcium-containing substrates were prepared inside a multi-well plate^24,30^. The test ink contained raw-alginate and wild-type cells to enhance the quality of later image acquisition. Dispensing was set at one minute per well. Medium was added right after printing. After four hours of incubation, the samples were stained with Calcein AM (living cells) and Propidium Iodide (dead cells). After fluorescent microscopy imaging, the live and dead cell count was performed in ImageJ and statistics were evaluated in Excel.

### Cell Handling

All cells (GFP C2C12, Wild-type NIH 3T3 fibroblasts and Tomato NIH 3T3 fibroblasts) were cultured in DMEM containing 10% fetal bovine serum in standard conditions: 37 °C, 5% CO_2_, 100% humidity. To prepare the bioinks, cells were rinsed twice with 1X PBS then treated with trypsin (0.25% for 5 minutes). Later cells were re-suspended in EDTA-PBS (1×) at high concentration (10E6 cells/mL) to be added to the bioinks.

### Statistics

A paired two tailed student’s t test was used. A p-value below 5% was considered as significant.

## Electronic supplementary material

Supplementary Figures
