# Supplementary material for: 3D Inkjet Printing of Complex, Cell-Laden Hydrogel Structures
Source: Sci Rep. 2018 Nov 20;8:17099. doi: 10.1038/s41598-018-35504-2 (PMC6244156; doi:10.1038/s41598-018-35504-2)
Supplement: Supplementary file 1 — Supplementary Figures [file 41598_2018_35504_MOESM1_ESM.pdf]

Supporting Information

3D Inkjet Printing of Complex, Cell-Laden Hydrogel Structures

*Andrea Negro, Thibaud Cherbuin and Matthias P. Lutolf**

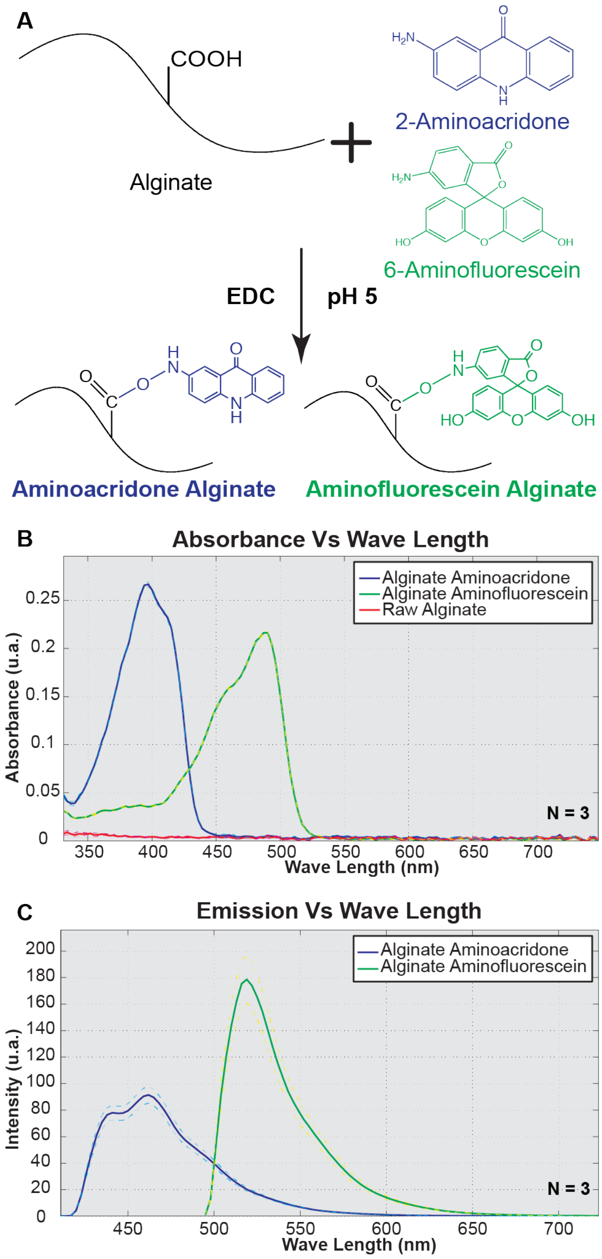

Figure S1: Alginate Conjugation. (A) Conjugation reaction. Carboxyl group on alginate backbone was activated by EDC to allow the fluorophores to bind to it. (B) Absorbance Spectra. Absorbance represented the first assessing method for validating the reaction. Blue and green lines represent the conjugated polymers, while the red line corresponds to the raw material. (C) Emission Spectra. Spectra were reconstructed by confocal microscopy taking into account the absorbance results for setting the excitations.

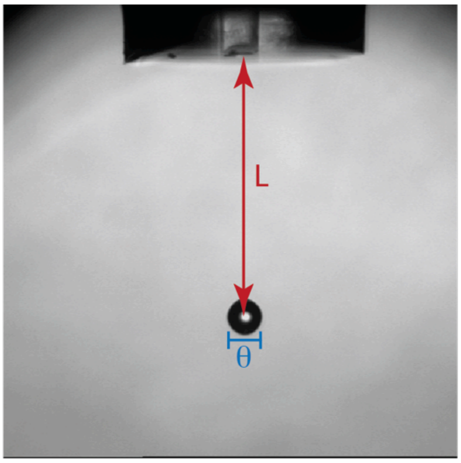

Figure S2: Scheme of the ejection state measurements. Droplet diameter (θ) and drop-nozzle distance (L) were measured for two different delays. Drop-nozzle distance data were later processed to obtain the ejection speed. Ejection speed was calculated by imaging the droplet formation phenomenon with two different delays and calculating the travel distanced between two time points.

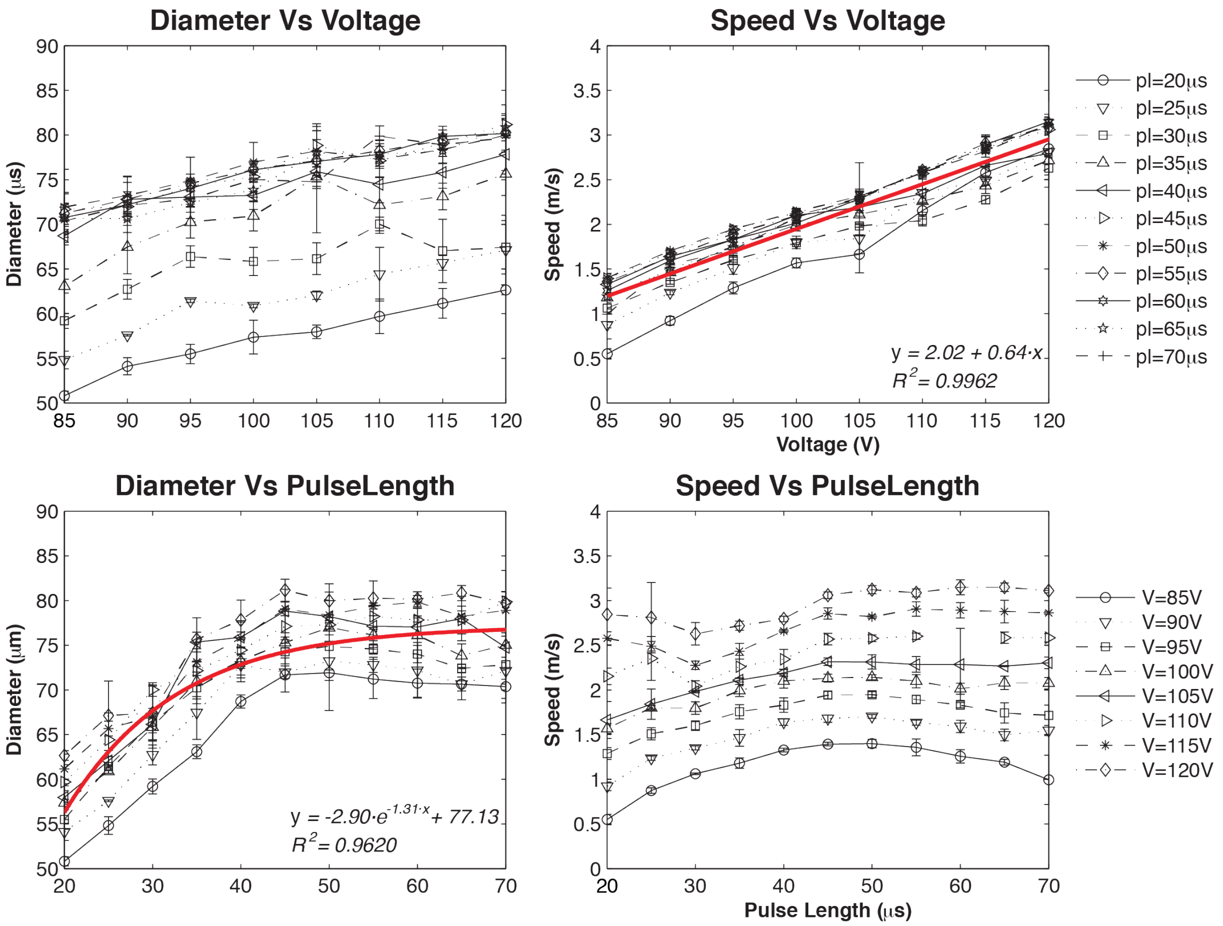

Figure S3: Ejection Stability – Acri-ink. All ejection data measured during ejection stability investigation. The main trends (red interpolating curve) are related to speed as a function of the voltage and diameter as a function of pulse length.

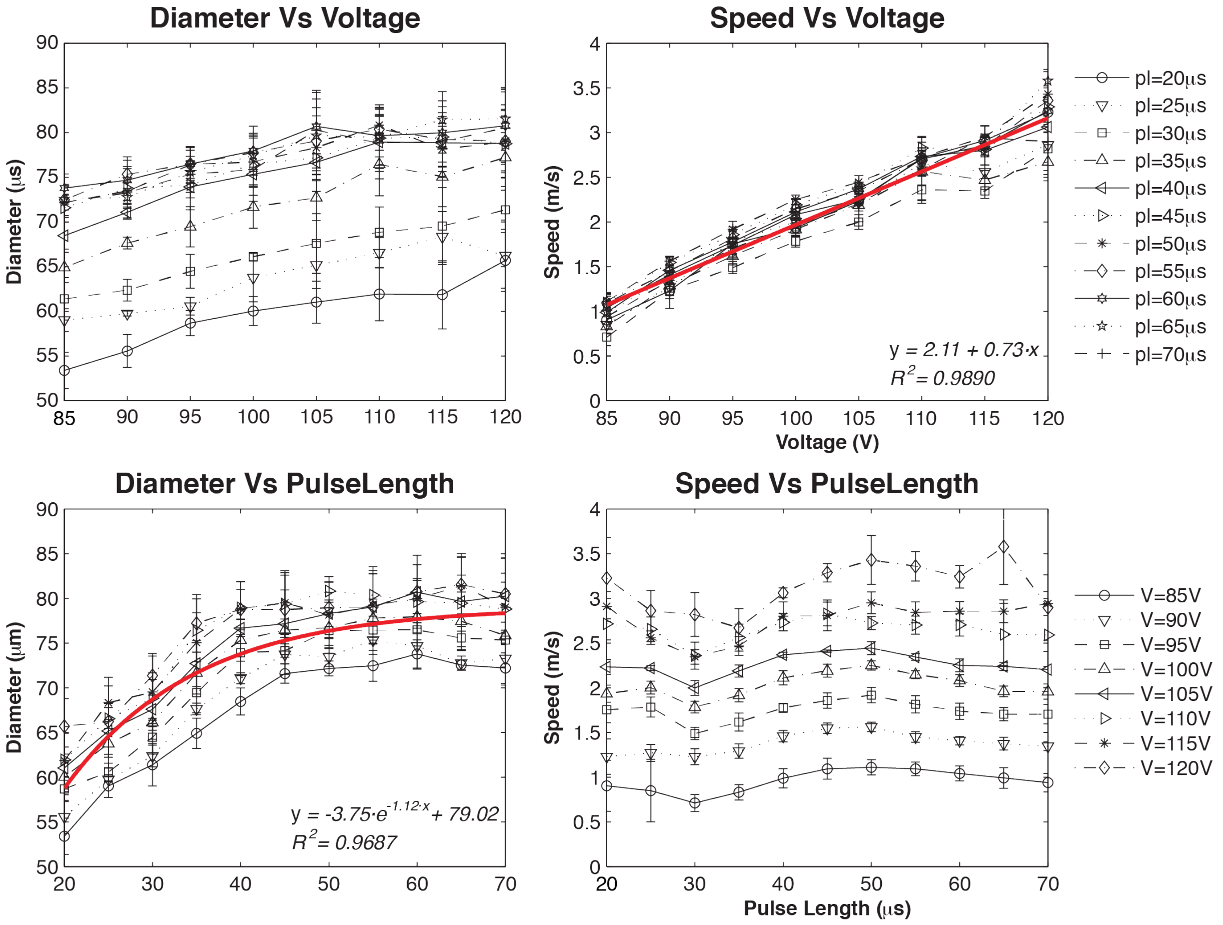

Figure S4: Ejection Stability – Fluo-ink. All ejection data measured during ejection stability investigation. The main trends (red interpolating curve) are related to speed as a function of the voltage and diameter as a function of pulse length.

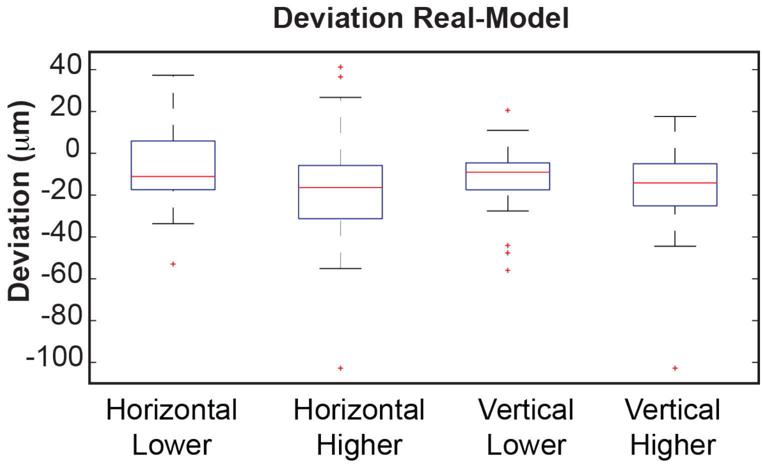

Figure S5: Deviation from model distributions. Samples were divided according to bottom versus top checkerboard, moreover the direction was taken in account (horizontal versus vertical).

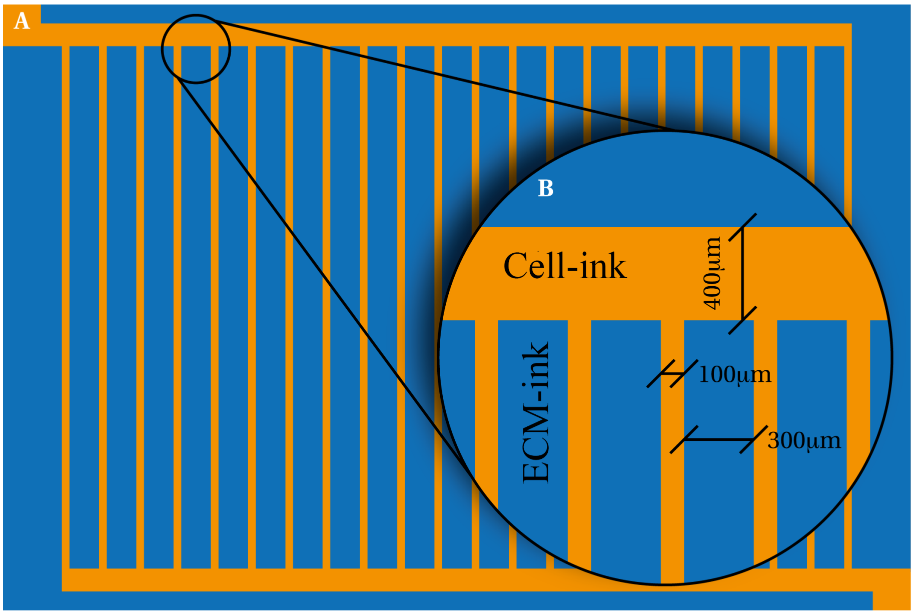

Figure S6: Ladder-like pattern. Pattern used to obtain a cellularized microfluidic network within the 3D printout. In blue, it is represented the ECM-ink. In orange, it is represent the Cell-ink. The square structures in (A), top-left and bottom-right, represent the inlet and outlet for the microfluidic chip. (B) A detail of the patter is reported, showing the fine geometric arrangement achievable with our technique. This pattern is printed between two structures of printed ECM-ink, which represent the ceiling and floor of the microfluidic channels.
